# A Weighted Gene Co-Expression Network Analysis–Derived Prognostic Model for Predicting Prognosis and Immune Infiltration in Gastric Cancer

**DOI:** 10.3389/fonc.2021.554779

**Published:** 2021-02-25

**Authors:** Qingchuan Chen, Yuen Tan, Chao Zhang, Zhe Zhang, Siwei Pan, Wen An, Huimian Xu

**Affiliations:** Department of Surgical Oncology, The First Affiliated Hospital of China Medical University, Shenyang, China

**Keywords:** gastric cancer, weighted gene co-expression network analysis, Cox proportional hazards regression model, prognostic model, immune infiltration

## Abstract

**Background:**

Gastric cancer (GC) is a major public health problem worldwide. In recent decades, the treatment of gastric cancer has improved greatly, but basic research and clinical application of gastric cancer remain challenges due to the high heterogeneity. Here, we provide new insights for identifying prognostic models of GC.

**Methods:**

We obtained the gene expression profiles of GSE62254 containing 300 samples for training. GSE15459 and TCGA-STAD for validation, which contain 200 and 375 samples, respectively. Weighted gene co-expression network analysis (WGCNA) was used to identify gene modules. We performed Lasso regression and Cox regression analyses to identify the most significant five genes to develop a novel prognostic model. And we selected two representative genes within the model for immunohistochemistry staining with 105 GC specimens from our hospital to verify the prediction efficiency. Moreover, we estimated the correlation coefficient between our model and immune infiltration using the CIBERSORT algorithm. The data from GSE15459 and TCGA cohort validated the robustness and predictive accuracy of this prognostic model.

**Results:**

Of the 12 gene modules identified, 1,198 green-yellow module genes were selected for further analysis. Multivariate Cox analysis was performed on genes from univariate Cox regression and Lasso regression analysis using the Cox proportional hazards regression model. Finally, we constructed a five gene prognostic model: Risk Score = [(-0.7547) * Expression (*ARHGAP32*)] + [(-0.8272) * Expression (*KLF5*)] + [1.09 * Expression (*MAMLD1*)] + [0.5174 * Expression (*MATN3*)] + [1.66 * Expression (*NES*)]. The prognosis of samples in the high-risk group was significantly poorer than that of samples in the low-risk group (p = 6.503e-11). The risk model was also regarded as an independent predictor of prognosis (HR, 1.678, p < 0.001). The observed correlation with immune cells suggested that this risk model could potentially predict immune infiltration.

**Conclusion:**

This study identified a potential risk model for prognosis and immune infiltration prediction in GC using WGCNA and Cox regression analysis.

## Introduction

Worldwide, gastric cancer (GC) is a common malignant tumor with relatively poor prognosis. The National Central Cancer Registry of China reported an estimated 679,100 new GC cases and 498,000 GC related deaths in 2015, making GC second in both cancer-specific incidence and mortality (1). There are a large number of patients with GC in China, the majority of whom have advanced stage disease. Over the past two decades, the 5-year overall survival of patients with GC has improved (2). This change is due to increased knowledge about the pathogenesis of GC, and treatment advances (3, 4). Such advances include the identification of GC biomarkers and therapeutic targets (5, 6). However, the limitations of surgery and cytotoxic chemotherapy mean that it is necessary to identify novel diagnostic and prognostic GC biomarkers.

We performed weighted gene co-expression network analysis (WGCNA) to identify gene modules related to the Asian Cancer Research Group (ACRG) GC molecular subtypes, Lauren subtypes, and other clinical traits using microarray data from the Gene Expression Omnibus (GEO) database. Next, we used Cox regression analysis to identify a prognostic model. Lastly, we evaluated the correlation between the generated prognostic risk model and tumor immune infiltration. Our data may offer novel insight in the search for prognostic biomarkers and the development of a predictive tool for GC.

## Materials and Methods

### Clinical Samples and Data Acquisition

**Figure 1:**
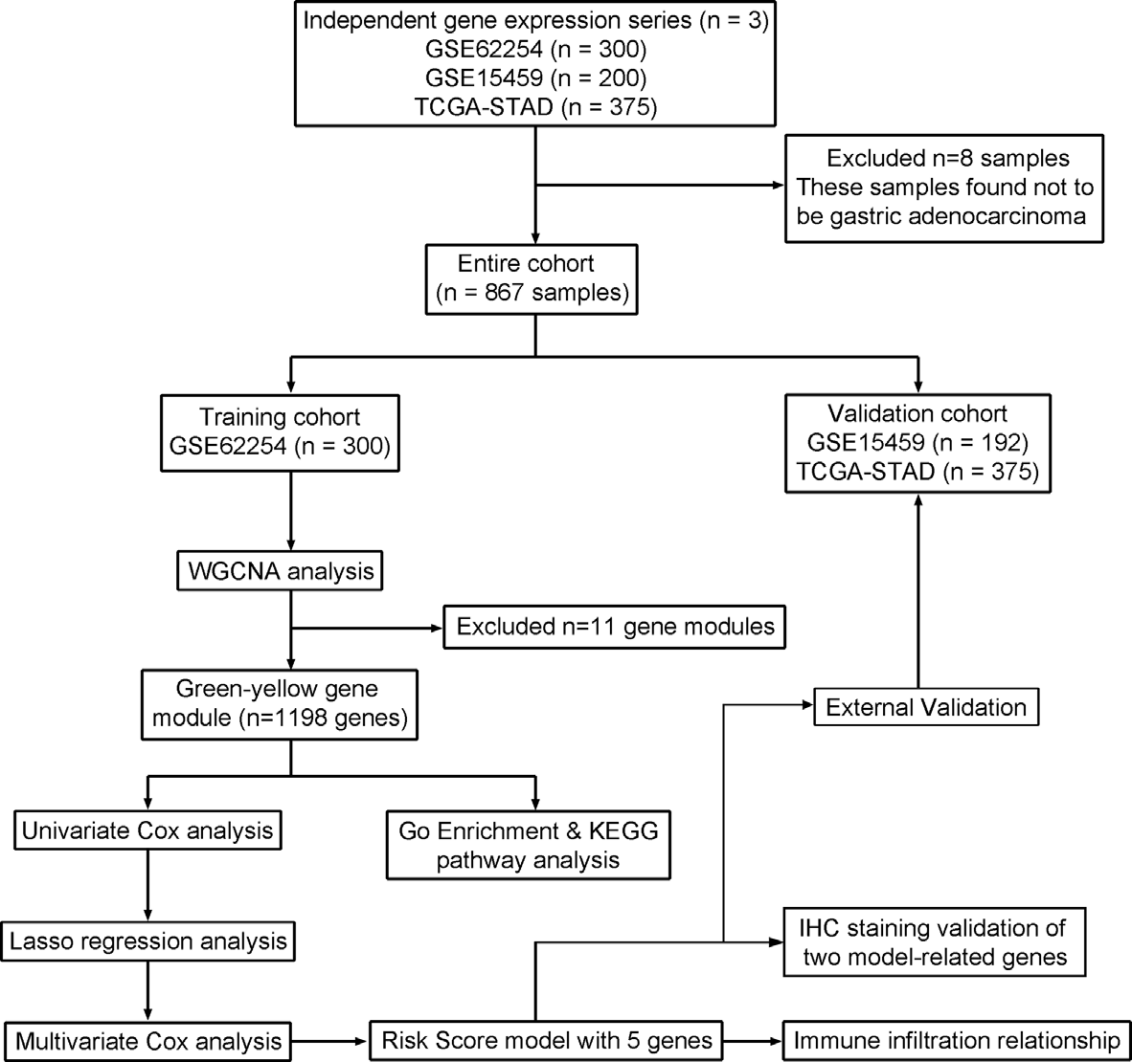
Flow chart of data acquisition, analysis, and validation.

We systematically searched the GC datasets in the public database. Finally, this study contains 875 patients with GC. Two data sets were retrieved from the GEO database, including 300 samples from the GSE62254 dataset (https://www.ncbi.nlm.nih.gov/geo/query/acc.cgi?acc=gse62254) and 200 samples from the GSE15459 dataset (https://www.ncbi.nlm.nih.gov/geo/query/acc.cgi?acc=GSE15459). Another TCGA-STAD cohort contains 375 samples (https://portal.gdc.cancer.gov). The GSE62254 microarray value was log10-transformed RMA signal intensity. The GSE15459 microarray was log10-transformed MAS5.0 signal intensity. And TCGA-STAD sequence data was FPKM values. We chose to use the GSE62254 dataset to construct a risk model because of the larger sample size and complete follow-up information, while the 575 cases from the GSE15459 dataset and TCGA-STAD cohort were used for model validation (**Figure 1**).

Where available, corresponding clinical data were extracted and manually organized. The GSE62254 clinical data was obtained from the primary literature **Supplementary Materials**, and the clinical data of GSE15459 and TCGA-STAD cohort were directly downloaded from the corresponding website in the GEO and TCGA database respectively.

To further validate the prognosis with real patient sample data, we obtained tumor specimens of 105 patients with GC who underwent radical surgery at the Department of Surgical Oncology, First Affiliated Hospital of China Medical University from 2003 to 2010. All patients did not receive preoperative chemotherapy and radiotherapy, and they were pathologically confirmed as gastric cancer. The resected tissue was fixed by formalin and embedded in paraffin for preservation. All patients have complete clinical, pathological, and follow-up data. This study was approved by the Ethics and Indications Committee of China Medical University, and all patients provided written informed consent.

### Data Processing

We obtained RNA expression profiles by downloading series matrix files in GEO and FPKM values in TCGA database. Data were normalized by Robust Multi-array Average (RMA) or Microarray Suite version 5.0 (MAS 5.0) using default Affymetrix analysis settings. We removed genes that were not expressed in all samples and used median values for those that were duplicated. As a result, data for 19572 genes was obtained from GSE62254. Genes with expression variances in the top 25% (n = 4893) were selected for subsequent WGCNA analysis.

### WGCNA

WGCNA is a systematic bioinformatic method, whose core algorithm is based on a weighted network and co-expression network. The regulatory relationship between genes is very complex and usually many-to-many. In a regulatory network, there are always a few highly-connected genes, called hub genes. WGCNA can simplify the interactions of thousands of genes into several modules of genes, whose expression pattern is similar, and changed highly coordinately. And then we calculate the network connection strength between any two genes, called weight value. WGCNA constructs a free-scale network through weighted and co-expression methods to explore the interrelationship between genes within the corresponding gene module, and the associations between gene modules and clinical traits. So, we choose WGCNA to filter out the junk information, to find the hub genes that met our expectations.

### Construction of the Weighted Correlation Network

After removing eight outliers by sample hierarchical clustering with the standard R function “hclust”, we constructed a weighted correlation network using the WGCNA package in R. The analysis was performed as described previously (7).

An adjacency matrix (a_ij_) was calculated by co-expression similarity (s_ij_). The a_ij_ represents the network connection strength between genes i and j, and s_ij_ is defined as the absolute value of the correlation coefficient between the expression profiles of genes i and j. The topological overlap matrix (TOM) calculated from a_ij_, suggests a neo-distance between those genes. The formulas used were:

$$s_{ij}=|cor(x_{i},x_{j}),\;a_{ij}=power(s_{ij},\beta )=s_{ij}\beta$$

$$TOM_{ij}=\frac{\Sigma_{u\neq i,j}(a_{iu}*a_{uj})-a_{ij}}{\min\{\Sigma_{u\neq i}a_{iu},\Sigma_{u\neq j}a_{uj}\}+1-a_{ij}}$$

In our study, the soft power of β = 3 (scale-free R^2^ = 0.903) was set as the soft threshold for the scale-free network. Soft threshold selection depends on scale independence and mean connectivity. Subsequently, the co-expression network clusters genes with similar expression patterns within the same module using Dynamic Branch Cut methods. We merged modules with highly correlated eigengenes, with a minimum module merging height of 0.2. Correlations between clinical traits and gene modules were displayed in a heatmap plot. Modules with the highest correlation coefficient and the lowest significant p-value were chosen for further analysis. To validate stability, we calculated the gene significance and module membership. Cytoscape 3.7.1 software was used to visualize significant module and hub genes using cytoHubba (8) and MCODE (9).

### Prognostic Model Construction Using the Cox Proportional Hazards Regression Model

A total of 1,198 genes of the hub module (the green-yellow module), with corresponding overall survival, were analyzed by univariate Cox survival analysis. This analysis revealed 476 significant genes (p < 0.01). The glmnet package from R was used for Least Absolute Shrinkage and Selection Operator (LASSO) Cox regression analysis (1,000 iterations) to reduce candidate variables and prevent overfitting (10). Nine genes from LASSO analysis were analyzed by multivariate Cox proportional hazards regression using the survival R package. The risk model was completed, and genes were divided into high- and low-risk groups based on the mean risk score. The formula of the risk score model is:

$$riskscore=\sum_{i=0}^{n}\gamma_{i}*\chi_{i}$$

where γ_i_ refers to the regression coefficient for each gene in the multivariate Cox hazard model analysis and χ_i_ represents the mRNA expression of the corresponding genes.

The prognostic model was validated using the GSE15459 dataset. For all above statistical analyses the significance level was p < 0.01.

### Functional and Pathway Enrichment Analysis

Gene Ontology (GO) and Kyoto Encyclopedia of Genes and Genomes (KEGG) pathway analyses were performed to identify the potential biological functions of the 476 genes identified using univariate cox analysis from the R package clusterProfiler, with the following parameters: pvalueCutoff = 0.05, qvalueCutoff = 0.05 (11).

### Immunohistochemistry

To explore the role of the model-related genes in GC tissue, we performed immunohistochemistry (IHC) staining for two genes with the highest absolute value of the coefficient in the risk formula.

IHC staining was performed in steps as the standard protocol (12, 13). Sections were deparaffinized using xylene and hydrated through an ethanol gradient. Incubation in 3% H2O2 for 20 min to inactivate endogenous peroxidase. After antigen retrieval using 0.01 mol/L sodium citrate buffer for 1.5 min at high pressure and 10% normal goat serum blocking for 30 min, sections were incubated with a primary antibody against *NESTIN* (1:100, Abcepta, San Diego, CA, USA), *KLF5* (1:200, Proteintech, Rosemont, IL, USA) at 4°C overnight. Subsequently, sections were incubated with secondary goat anti-rabbit antibody for 30 min and counterstained with hematoxylin. We use the product of two scores based on the percentage of positive cells (0: <5%, 1: 5%–25%, 2: 25%–50%, 3: 50%–75%, and 4: >75%) and the staining intensity (0: negative, 1: weak, 2: moderate, and 3: strong) to evaluate IHC staining. We regarded the scores of 6–12 as high expression, while the scores of 0-4 were low expression.

### CIBERSORT and TIMER

The LM22 gene signature matrix and CIBERSORT algorithm can estimate the relative proportions of 22 human immune cell phenotypes, including T cells, B cells, NK cells, macrophages, DC cells, mast cells, and granulocytes, in complex bulk tumor tissue (14). CIBERSORT adaptively selects genes from the input matrix to deconvolve a given mixture using linear support vector regression (SVR) based on the LM22 signature matrix. The LM22 was validated using external datasets of each cell subset, and CIBERSORT results were well-matched (93%) with the phenotypes of these datasets (14). The input matrix of reference gene expression signatures was prepared using the standard annotation file. The CIBERSORT algorithm runs in R with 100 permutations using the LM22 signature, and p < 0.05 was set as the cutoff for statistically significance. TIMER algorithm can estimate the abundances of six immune infiltrates and evaluate the correlation of gene expression with immune infiltration level (15).

### Statistical Analysis

The Levene’s test was used to test whether the variances of two or more independent samples are equal. The Shapiro-Wilk normality test was used to determine the normality of variables. When there were two sets of variables, the unpaired student t-test was used to compare variables with normal distribution, and the Mann-Whitney U test (also called the Wilcoxon rank-sum test) was used to compare variables with non-normal distribution. The Kruskal-Wallis test was used as a non-parametric test to estimate the statistical differences between multiple groups of variables. The Spearman correlation test was employed to estimate the correlation between two non-normally distributed continuous variables.

The Kaplan-Meier survival curve was generated using the R package “survival” and “survminer”. The R package “timeROC” was used to plot time-dependent receiver operating characteristic (ROC) curves and calculate the area under the curve (AUC), which evaluates the diagnostic accuracy of risk-score, stage, and the combination of risk-score and stage.

The hazard ratios for univariate and multivariate analyses were calculated using the Cox proportional hazards regression model. A multivariate Cox regression model was used to determine independent prognostic factors. All Cox regression was performed by R package survival, and the glmnet package was used for LASSO Cox analysis. All statistical analyses were conducted using R 3.6.2 and SPSS 26.0 software, and p < 0.05 was considered statistically significant.

## Results

### Pre-Processing of RNA Sequence Data and Clinical Data

In total, GC microarray and clinical data from 500 patients were downloaded from the GSE62254 and GSE15459 datasets. For the training cohort, the RNA sequence expression matrix from GSE62254, composed of 19572 genes from 300 patients, after removal of abnormal and duplicate values. Genes with expression in the first quarter of variance was selected for further WGCNA analysis.

### Identification of Modules Associated With Clinical Traits by WGCNA

**Figure 2:**
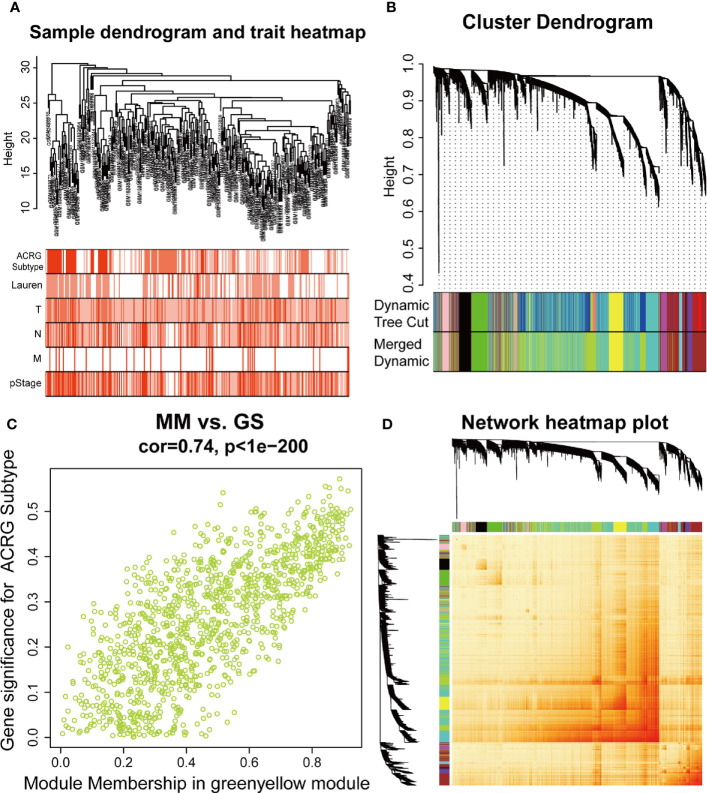
Identification of the weighted gene co-expression network analysis (WGCNA) hub module and relationship with clinical traits. **(A)** Sample cluster dendrogram and heatmap of corresponding clinical traits. Each branch represents a sample of gastric cancer (GC). **(B)** Dendrogram of genes clustered into 12 colored modules based on a dissimilarity measure (1-TOM). Each branch represents a gene. **(C)** The correlation between green-yellow module and Asian Cancer Research Group (ACRG) subtype. MM, module membership; GS, gene significance. **(D)** The cluster dendrogram and heatmap of network based on the topological overlap matrix.

**Figure 3:**
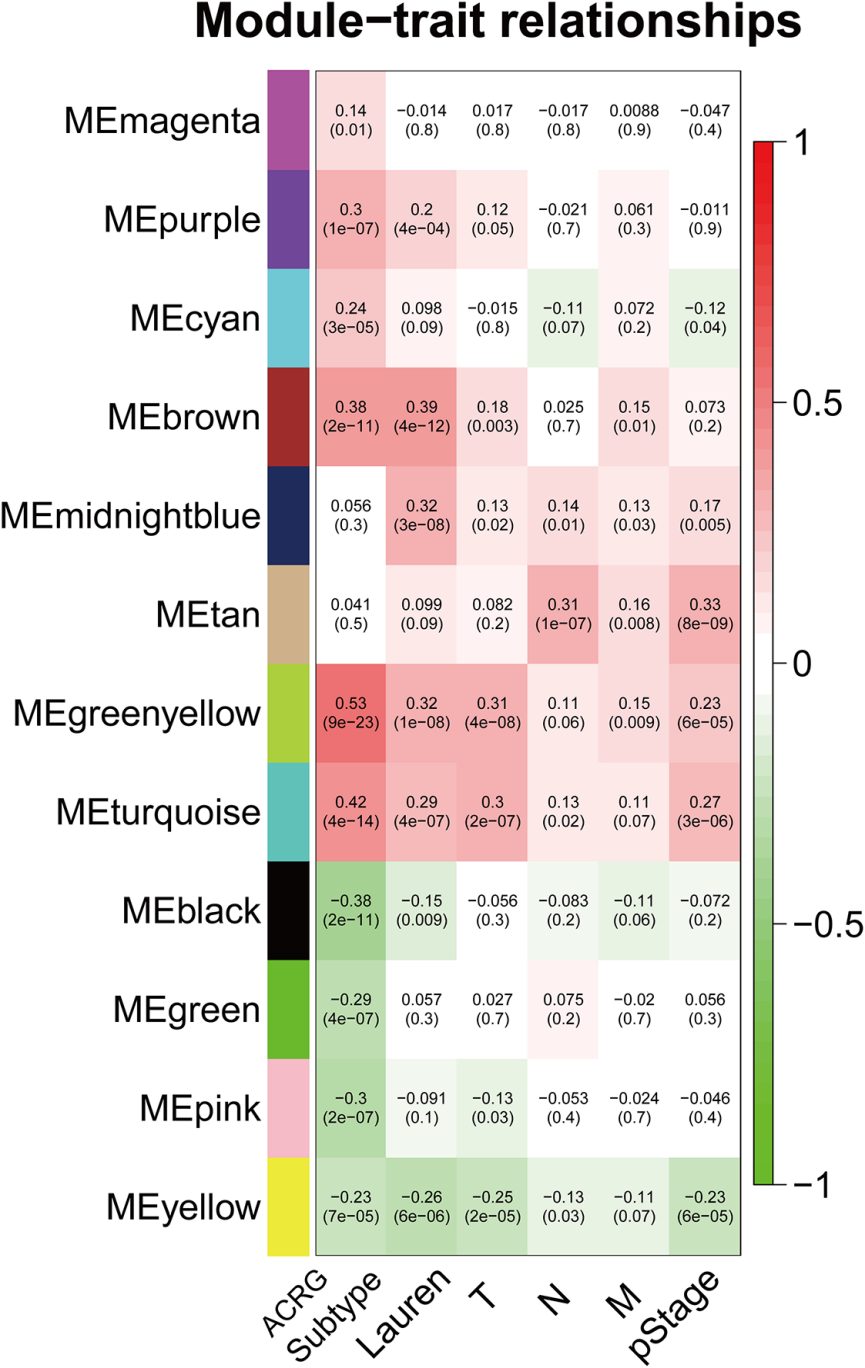
Heatmap of the correlation between MEs and clinical traits. The background colors of the cells represent the strength of correlations, from red to green. The numbers in the cells represent the correlation coefficient, and the numbers in parentheses represent p-values. ME, module eigengene.

The sample dendrogram and trait heatmap described the clustering landscape of GSE62254 sample (**Figure 2A**). After sample hierarchical clustering, eight outliers were removed (**Figure S1A**), and the soft threshold was set as β = 3 (scale-free R^2^ = 0.903) based on scale independence and mean connectivity (**Figure S1B**). We merged modules with similarity above 0.8 (**Figure S1C**). Finally, the dynamic tree cut showed a gene cluster dendrogram containing 12 co-expression models (**Figure 2B**). The co-expression models are represented by black, brown, cyan, green, green-yellow, magenta, midnight-blue, pink, purple, tan, turquoise, and yellow and contain 215, 745, 63, 293, 1198, 92, 37, 116, 91, 69, 1527, and 447 genes, respectively. The scatter diagram illustrated the relationship between the gene significance (GS) for ACRG subtype and the module membership (MM) in green-yellow module, with a correlation coefficient of 0.74 (**Figure 2C**). We constructed a cluster dendrogram and heatmap based on the topological overlap matrix (**Figure 2D**). The module-trait relationship also revealed that the green-yellow module was strongly related to clinical traits, especially the ACRG molecular subtype (**Figure 3**). Based on these analyses, we considered the “green-yellow” module, containing 1198 genes, the target module for further analysis.

### Visualization of the Module Genes With Cytoscape

**Figure 4:**
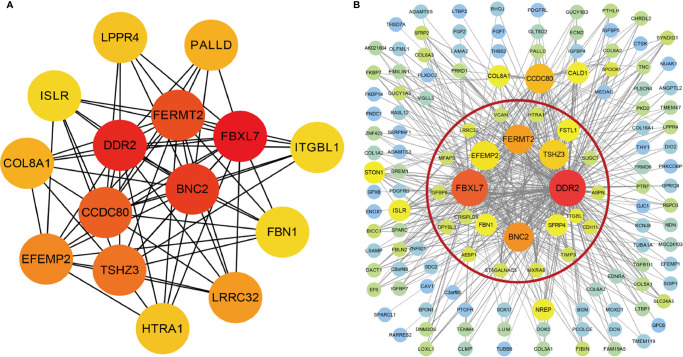
Visualization of the co-expression genes of green-yellow module in Cytoscape. **(A)** The network containing the top 15 genes ranked by Maximal Clique Centrality (MCC) algorithm of cytoHubba plugins. **(B)** The top 500 green-yellow module genes based on weight values. The gene module inside red circle was identified by MCODE plugins.

The interrelationship (edges) of all 1,198 genes (nodes) were too complex to comprehensively visualize in Cytoscape, so we selected the top 500 genes based on the weight values of the nodes. The network was constructed by cytoHubba and MCODE plugins. cytoHubba contained 11 scoring methods, including the newly developed algorithm named Maximal Clique Centrality (MCC). We selected the top 15 hub genes ranked by the MCC (**Figure 4A**), and MCODE identified one key module containing 24 hub genes from the network (**Figure 4B**). A total of ten genes were identified using the methods: *FBXL7*, *DDR2*, *BNC2*, *FERMT2*, *TSHZ3*, *EFEMP2*, *LRRC32*, *HTRA1*, *ITGBL1*, and *FBN1*.

### GO Enrichment and KEGG Pathway Analysis

**Figure 5:**
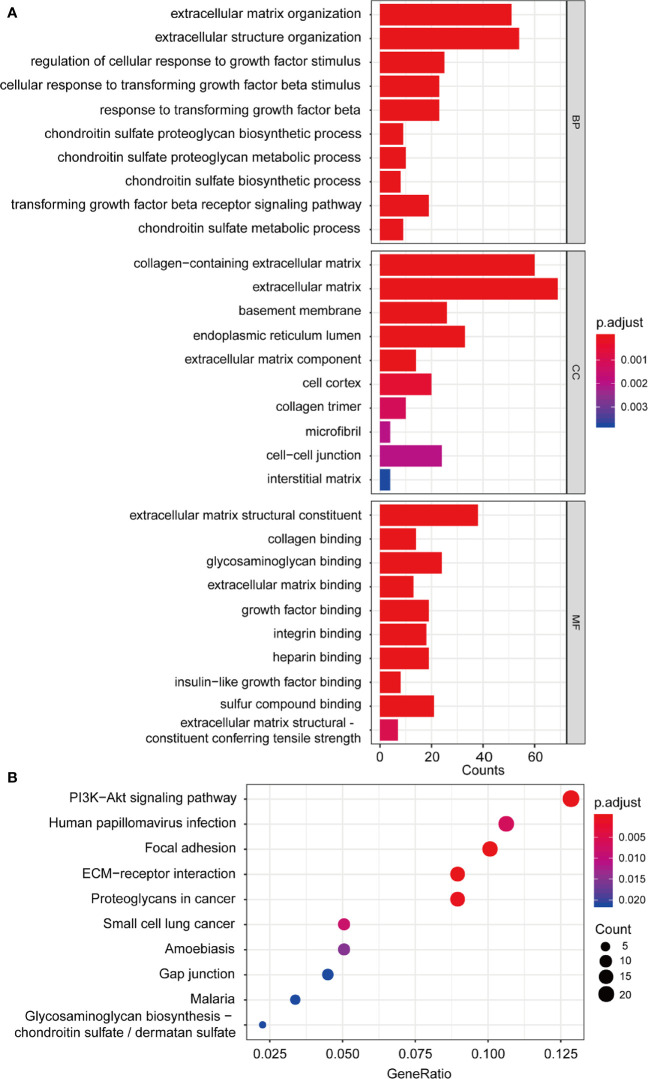
Functional enrichment analysis. **(A)** GO enrichment analysis including biological process, cellular component, molecular function analysis; **(B)** KEGG pathway enrichment analysis. GO, Gene Ontology; KEGG, Kyoto Encyclopedia of Genes and Genomes.

Univariate Cox regression was performed with 1,198 genes from the green-yellow module, and showed that 476 genes were significantly related to overall survival (**Table S1**). To identify the functional categories and biological pathways of these genes, we performed GO enrichment and KEGG pathway analysis using the “clusterProfiler” of R package. The enriched biological process was mainly involved in the extracellular matrix (ECM), TGF-β, and chondroitin sulfate. The enriched cellular components were mainly the extracellular matrix, basement membrane, and endoplasmic reticulum lumen. The molecular functions mainly included extracellular matrix structural constituents and types of protein binding. The KEGG pathway analysis revealed enrichment in ECM-receptor interaction, focal adhesion, PI3K-Akt signaling pathway, and proteoglycans in cancer (**Figures 5A, B**).

### Construction and Validation of the Cox Regression Model

**Figure 6:**
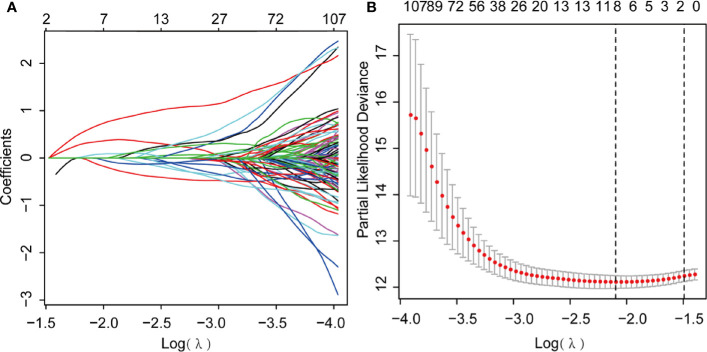
Least Absolute Shrinkage and Selection Operator (LASSO) Cox regression analysis. **(A)** LASSO coefficient profiles; **(B)** Genes from univariate Cox regression analysis were narrowed down by the lasso algorithm.

We further performed LASSO regression analysis using “glmnet” from the R software package. First, we analyzed the trajectory of each independent variable, shrinking some coefficients and setting others to zero. Cross-validation was also employed for model construction, and the confidence interval under each lambda is presented in **Figure 6A**. The candidate genes were narrowed down to eight genes with lambda = 0.1287 (**Figure 6B**). Then, the multivariate Cox regression analysis was applied further. Finally, we identified five genes (**Table 1**) for the prognostic model:

Risk Score=[(−0.7547)∗Expression (ARHGAP32)+[(−0.8272)∗Expression (KLF5)]+[1.09∗Expression (MAMLD1)]+[0.5174∗Expression (MATN3)+[1.66∗Expression(NES)]

**Table 1:** The multivariate Cox regression analysis.

Gene symbol	Coefficient	HR	Low 95% CI	High 95% CI	P value
ARHGAP32	−0.7547	0.4702	0.1828	1.2095	0.1175
KLF5	−0.8272	0.4373	0.2018	0.9476	0.0360
MAMLD1	1.0900	2.9743	1.0344	8.5517	0.0431
MATN3	0.5174	1.6776	1.0210	2.7567	0.0412
NES	1.6600	5.2595	1.9137	14.4552	0.0013

We then used the median risk score to divide the 292 patients into high risk (n = 146) and low risk (n = 146) groups.

**Figure 7:**
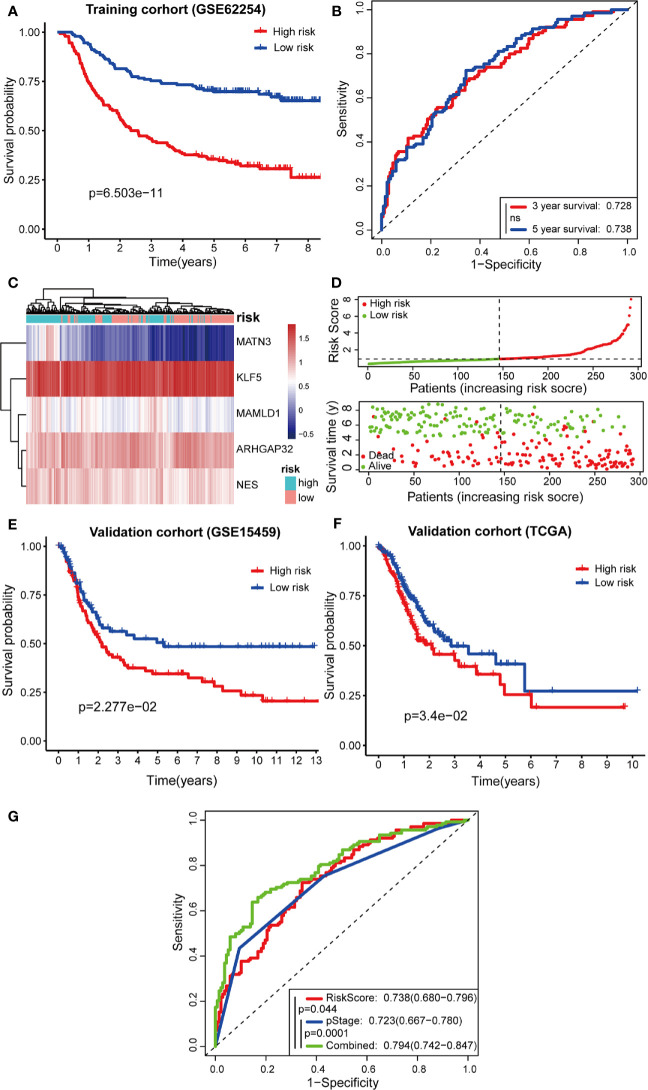
Predictive effects of prognostic models. **(A, B)** Kaplan-Meier curves and receiver operating characteristic (ROC) curves of GSE62254; **(C, D)** High- and low-risk group gene expression heatmap, Risk Score rank, and survival status distribution; **(E, F)** Kaplan-Meier curves of GSE15459 and TCGA cohort; **(G)** Comparison of ROC curves between Risk Score model, pStage, and Combined model.

Next, Kaplan-Meier curves (**Figure 7A**) showed that our predictive model was significant using both the high-risk and low-risk groups (p = 6.503e-11). Patients in the high-risk group were considered to have a poor prognosis, while patients in the low-risk group seemed to have a better prognosis. Through the risk coefficient obtained by the formula, we predicted the survival probability of each sample and compared it with the actual survival probability of patients (3 or 5 years). We drew the ROC curve and got the AUC value using SurvialROC method in R. The 3- and 5-year area under the ROC curve were 0.728 and 0.738, respectively, suggesting a moderate potential for the prognostic signature in survival monitoring (**Figure 7B**). Focusing on the distribution of the survival status of these two groups, we can observe the stratification of survival status. The heatmap of five model genes showed that *MATN3*, *MAMLD1*, and *NES* were highly expressed in the high-risk group, and that *KLF5* and *ARHGAP32* were expressed at low levels in the low-risk group (**Figures 7C, D**).

To confirm the robustness of the prognostic model, the same gene expression formula was used to analyze the GSE15459 dataset and TCGA cohort. Kaplan-Meier curves (**Figures 7E, F**) showed that patients of GSE15459 with low-risk had an obviously better 5-year survival rate than did those with high-risk (p = 0.02277); the same with the condition in TCGA cohort (p = 0.034). These results suggest that the prognosis was significantly better for patients in the low-risk group than for those in the high-risk group.

Furthermore, the TNM levels, including depth of tumor invasion, regional lymph nodes, and metastatic diseases, were incorporated into our prognostic model to construct a combined model. According to the ROC painted by the R package “SurvivalROC”, we accessed the prediction efficiency of this model. The AUC of the “Risk Score”, “pStage”, and “combined” three models, was 0.738, 0.723, and 0.794, respectively (**Figure 7G**). The ROC of the combined model was significantly higher than that of the other two models.

**Figure 8:**
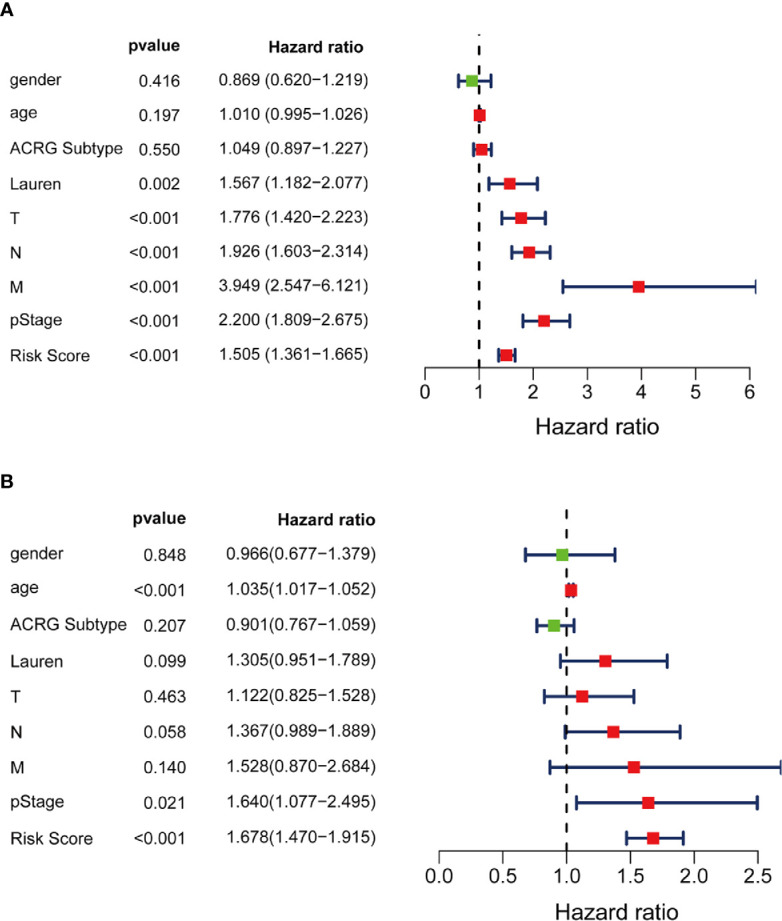
Independent prognosis effect of Risk Score model. **(A)** univariate Cox regression analysis; **(B)** multivariate Cox regression analysis.

Moreover, multivariate Cox stepwise proportional hazards analysis identified clinical traits pStage (HR, 1.64, p = 0.021), and risk-score (HR, 1.678, p < 0.001) as independent predictors of prognosis. Although Lauren subtypes, T, N, M, and pStage were regarded as independent prognostic factors using univariate analysis, only pStage and risk-score were significant in the multivariate analysis (**Figures 8A, B**).

### IHC Staining of 105 Patients

**Figure 9:**
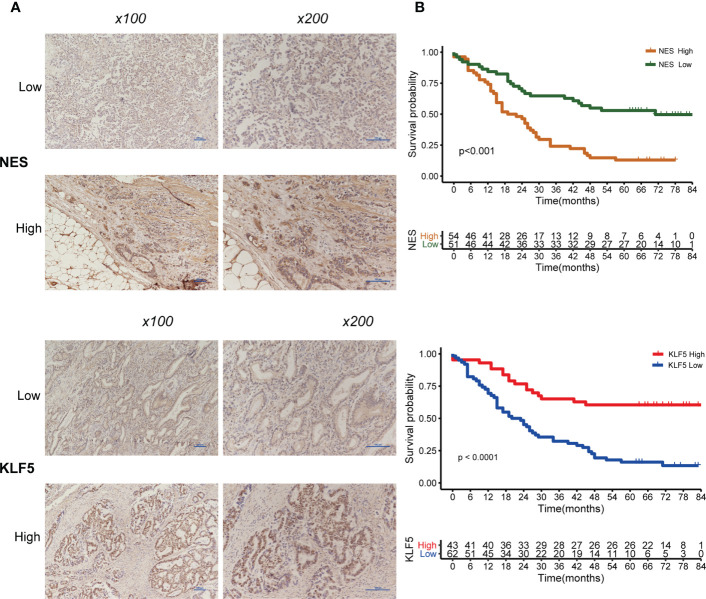
Expression of NES and KLF5 in gastric cancer (GC) tissue and Kaplan-Meier analysis. **(A)** Low and high expression of NES and KLF5 in GC specimens; **(B)** Kaplan‐Meier survival curves based on NES and KLF5.

Of the IHC analysis of 105 patients with GC, we found that *NES-* and *KLF5-*positive expression rates were 51.4% (54/105) and 41.0% (43/105), respectively (**Figure 9A**). Survival analysis suggested that patients with high expression of *NES* had a worse prognosis (p < 0.001), while the high expression of *KLF5* associated with a better prognosis (p < 0.001, **Figure 9B**).

### Clinical Relevance of the Prognostic Model

**Figure 10:**
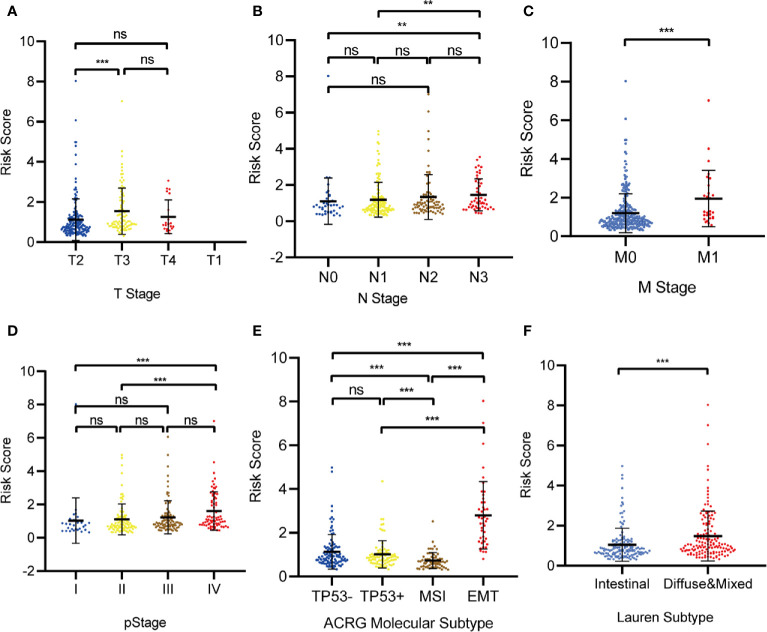
The correlation between the Risk Score and **(A)** T stage; **(B)** N stage; **(C)** M stage; **(D)** pStage; **(E)** Asian Cancer Research Group (ACRG) molecular subtype; **(F)** Lauren subtype. (*p < 0.05, **p < 0.01, ***p < 0.001, ns, no significance).

Relationships between the prognostic model risk score and clinical and demographic characters, including gender, age, T, N, M, pStage, Lauren subtype, and ACRG molecular subtype were analyzed. Our results show that the risk score was significant with T, N, M, pStage, Lauren subtype, and ACRG molecular subtype. The test statistics and p values are shown in the following table (**Table 2**). Our results show that the risk score of this model was significantly correlated with clinical traits, such as T stage, N stage, M stage, pStage, Lauren subtype, and ACRG molecular subtype. However, no risk score differences were observed for gender and age (**Figures 10A–F**).

**Table 2:** The relationships between the risk score and clinical traits.

	Variables	P value
Risk Score	Gender^1^	0.630
	Age^1^	0.136
	T Stage^2^	0.000
	N Stage^2^	0.011
	M Stage^3^	0.000
	pStage^2^	0.000
	ACRG Subtype^2^	0.000
	Lauren Subtype^1^	0.001

^1^Unpaired Student t-test.

^2^Kruskal-Wallis H Test.

^3^Mann-Whitney U Test.

### Immune Infiltration of the Five Identified Genes and Model Risk Score

**Figure 11:**
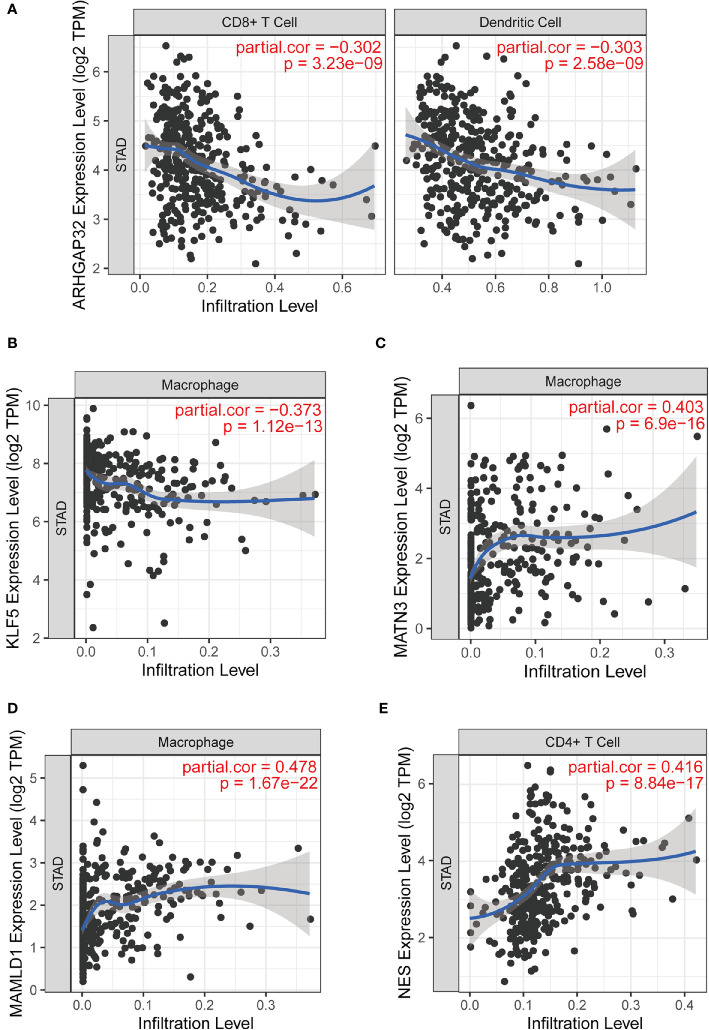
Part of relationships between model genes and immune cells evaluated by TIMER. **(A)** ARHGAP32 with CD8+ T cell and dendritic cell; **(B)** KLF5 and macrophage; **(C)** MATN3 and macrophage; **(D)** MAMLD1 and macrophage; **(E)** NES and CD4+ T cell.

We analyzed the relationship between the prognostic model genes and six immune cell infiltration using the TIMER algorithm. All five genes were significantly related to immune cells (**Figures S2–S6**), especially CD8+ T cells, CD4+ T cells, macrophages, and dendritic cells. *ARHGAP32* was negatively related to CD8+ T cells and dendritic cells, *KLF5* was negatively related to macrophages, *MATN3* and *MAMLD1* were highly positively related to macrophages, and *NES* was positively related to CD4+ cells (**Figures 11A–E** **)**.

**Figure 12:**
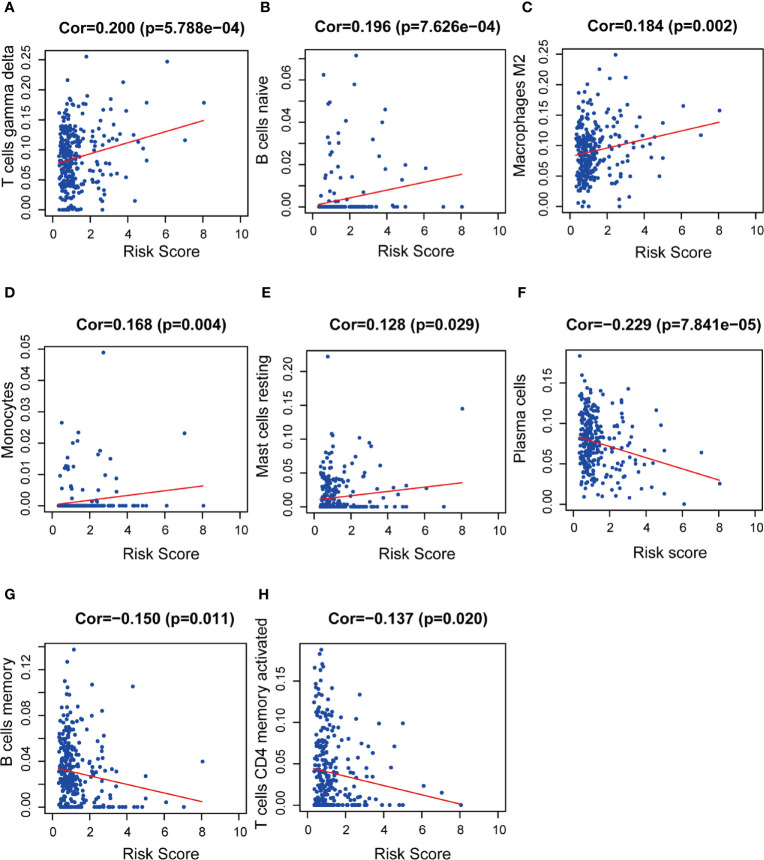
The relationships between the prognostic model and immune infiltration estimated by CIBERSORT. The correlation was performed by using Pearson correlation analysis. **(A)** T cells gamma delta; **(B)** naïve B cells; **(C)** M2 macrophages; **(D)** monocytes; **(E)** resting mast cells; **(F)** plasma cells; **(G)** memory B cells; **(H)** CD4+ memory activated T cells.

We used CIBERSORT to estimate the immune cell proportion in each patient sample, and to assess the relationship between the risk score and immune infiltration using Spearman correlation test. We found that B cell subsets, including plasma cells, naïve B cells, and memory B cells, and CD4 memory activated T cells was related to the risk score. Moreover, M2 type macrophages, monocytes, and activated NK cells were also significantly correlated with risk score. The results showed a negative correlation between the risk score and plasma cells, memory B cells, CD4+ memory activated T cells, and activated NK cells. M2 macrophages, naïve B cells, and monocytes are positively correlated with the risk score (**Figures 12A–H**).

## Discussion

Many genetic prognostic models of GC have been published, most of which are based on genetic difference analysis followed by Cox regression analysis. This study is based on weighted co-expression network and detailed gene set analyses to obtain meaningful modules and construct a prognostic model. The correlation between this model and immune infiltration was evaluated, and our results suggest a significant correlation between immune infiltration and prognosis in patients with GC.

Because of the high heterogeneity of GC, and the development of molecular detection technology, attention on the molecular classification of GC is increasing. We chose ACRG/GSE62254 for WGCNA analysis, because it contains a large number of ACRG cases (N = 300) and comprehensive clinical information and is divided into four widely recognized subtypes: MSI; MSS/TP53+; MSS/TP53-; and MSS/EMT. The green-yellow module was strongly related to the ACRG molecular subtype.

We selected the top 500 genes, based on edge weight coefficients, for Cytoscape visualization. We found 10 genes that were significant in both cytoHubba and MCODE plugins. These 10 genes have been shown to affect oncogenesis and tumor development through various mechanisms. For example, *DDR2* promotes GC peritoneal dissemination through collagen deposition by stromal fibroblasts in the microenvironment (16). Another report showed that *DDR2* mediates stromal and cancer cell interaction in mesenchymal stem cells and metastasis growth in breast cancer (17, 18). *EFEMP2* significantly inhibits the invasion and metastasis of tumor cells and the process of epithelial-mesenchymal transition (EMT) through the Wnt/β-catenin pathway in lung, bladder, and breast cancers (19–21). Similarly, *ITGBL1* is involved in tumor cell invasion and metastasis through the KRAS/EMT pathway (22). Extracellular vesicles enriched in *ITGBL1* can activate fibroblasts, which induce metastasis by secreting proinflammatory cytokines (23).

Tumor development and metastasis are multistep and complex processes that involve the interaction of the tumor microenvironment, composed of tumor cells and stromal cells (24). Functional enrichment analysis of the 476 genes revealed that ACRG molecular subtype was related to the ECM, chondroitin sulfate, and TGF-β for molecular functions. KEGG pathway analysis results revealed that PI3K-Akt signaling pathway, focal adhesion, ECM-receptor interaction, and glycosaminoglycan biosynthesis were valuable target pathways in GC pathogenesis research. All above characteristics are related to GC oncogenesis and metastasis mediated by the ECM and its receptor, integrins. The cancer-associated ECM, surrounding tumor and stromal cells, is a complex part of the tumor microenvironment that mainly contains collagen, proteoglycans (including chondroitin sulfate and heparan sulfate), ECM proteins (including MMPs), and other factors. In cancer cells, abnormal integrin activity promotes oncogenesis through ECM remodeling or by interfering with intracellular or extracellular signaling transduction (25). Integrin consists of 18 α and 8 β subunits. The PI3K/AKT pathway is preferentially activated in response to αvβ3 integrin, which inhibits tumor cell apoptosis by targeting the pro-apoptotic Bcl-2 related protein (25). The ECM can provide normal tissue/cells for structural support and signal transduction to maintain physiological activity, but the signaling cascades mediating cell-ECM interactions can remodel the ECM structure and function to promote the growth, adhesion, invasion, and migration of tumor cells.

We applied WGCNA to identify the hub modules and to select appropriate genes for further analysis. In this study, genes within the green-yellow module were selected to construct the gene prognostic model risk scoring system. Finally, we established a five gene prognostic model that was independently validated with an external dataset and shown to be accurate.

Of the five genes within the novel risk model, there have been no reports about the function or mechanism of *ARHGAP32* and *MAMLA1* in GC. The other three genes play important roles in the molecular mechanisms of GC progression. *Krüppel-like factor 5* (*KLF5*) is a zinc-finger transcription factor, which regulates cell growth, proliferation, differentiation, and tumorigenesis in several cancers, including GC. Kwak et al. found that *KLF5* expression increased in early GC, small GC, and N0 stage GC (26). These findings indicate patients with early stage GC, or without lymph node metastasis, may benefit from increased *KLF5* expression after GC surgery. These results suggest that high levels of *KLF5* expression may be related to a relatively better prognosis. However, Fujii et al. identified *KLF5* as a stemness-associated reprogramming factor (27). They also considered that *KLF5*, induced by *CDX1*, converts gastric epithelial cells to intestinal stem/progenitor-like cells, which have properties similar to those of cancer stem cells. Acquisition of stemness makes epithelial cells dedifferentiate and transdifferentiate to stem/progenitor cells. Chia et al. also found that *KLF5* expression can be induced by metaplasia-inducing factors such as *CDX1* and *Helicobacter pylori* infection. Moreover, *KLF5*, *GATA4*, and *GATA6* represent lineage-survival oncogenes in GC with a synergistic effect (28). In short, KLF5 is related to several pathological processes, including a neoplastic change in the stomach.

*MATN3* is a non-collagenous ECM component and induces the expression of MMPs, indicating that *MATN3* can regulate ECM degradation. Wu et al. found that high *MATN3* expression indicates a poor prognosis and is involved in the process of GC growth and metastasis (29).

*NES* (*Nestin*), a cytoskeleton-associated class VI IF protein, is a neuronal stem/progenitor cell marker expressed in progenitor cells of various tissues, including central nervous system tumors, lung cancer, and breast cancer. Recent reports support a link between NES and malignant characteristics and suggest that abundant NES expression is correlated with increased malignancy and poorer prognosis in different cancers. Moreover, NES regulates the EMT and malignant prognosis in GC (30).

Our WGCNA analysis-based prognostic signature demonstrates favorable clinical viability. Notably, our risk model shows moderate prognostic predictions and correlates with ACRG molecular subtype, Lauren subtype, T, N, M, and pStage. Furthermore, this risk model is related to the tumor microenvironment *via* the ECM. We performed correlation analysis to confirm the correlation between the model and immune cells. Immune infiltration conditions, using the TIMER website tool, showed that *KLF5* was negatively correlated with macrophages, while *MATN3*, *NES*, and *MAMLD1* were positively correlated with macrophages. *MAMLD1*, *NES*, *MATN3* were positively correlated with CD4+ T cell, and *KLF5* was negatively correlated with CD4+ T cells. Macrophages are one of the main components of the tumor immune microenvironment. Tumor-associated macrophages secrete a variety of cytokines, degrade and reconstitute the extracellular matrix, and promote tumor cell migration and invasion. M2 type macrophages were also recruited by tumor cells to suppress inflammatory and immune responses. CD4 + T cells play an important role in maintaining tumor immunity. Moreover, we identified a relationship between our risk model and additional immune cell subtypes. Our risk model was positively correlated with M2 macrophages, naïve B cells, and monocytes, and negatively correlated with CD4+ memory activated T cells, plasma cells, memory B cells, and activated NK cells. These results indicate that the higher infiltration levels of M2 macrophages, naïve B cells, and monocytes, and lower infiltration levels of memory activated CD4+ T cells, plasma cells, and memory B cells might be observed in high-risk patients. The data in this study suggests that this risk model is not only a potent predictor of prognosis in patients with GC but also has the potential to predict immune infiltration levels. However, the role of immune infiltration in the GC microenvironment remains unclear, and corresponding mechanisms need to be experimentally verified in the future.

This study was designed to identify a prognostic risk model of GC using WGCNA and Cox regression analysis. In summary, *ARHGAP32*, *KLF5*, *MAMLD1*, *MATN3*, and *NES* are considered hub genes of this risk model, and validation results using an external dataset show significantly different prognostic outcomes. The correlation between the risk model and immune infiltration suggests that hub genes may influence the tumor microenvironment through interacting with immune cells. This study was limited by the size of the validation dataset, and thus we were unable to further identify the reliability of this model. The predictive effects and specific mechanisms of this risk model require further research.

## Data Availability Statement

The data sets presented in this study can be found in online repositories. The names of the repository/repositories and accession number(s) can be found in the article/**Supplementary Material**.

## Ethics Statement

The studies involving human participants were reviewed and approved by ethics and indications committee of China Medical University. The patients/participants provided their written informed consent to participate in this study.

## Author Contributions

QC, YT, and HX: conception and design. QC, CZ, and ZZ: acquisition of data. QC, SP, and WA: analysis and interpretation of data. QC, YT, and HX: writing and review of the manuscript. All authors contributed to the article and approved the submitted version.

## Funding

This research was supported by the National Natural Science Foundation of China no. 81772549 and National Natural Science Foundation of China no. 81572334.

## Conflict of Interest

The authors declare that the research was conducted in the absence of any commercial or financial relationships that could be construed as a potential conflict of interest.

## Supplementary Material

Supplementary Figure 1
**(A)** Sample cluster dendrogram. Red line represents height of 31. **(B)** Analysis of scale-free independence and mean connectivity to select optimal soft-threshold power. **(C)** Clustering of MEs. The red line represented height of 0.2. ME, module eigengenes.

Supplementary Figure 2 -S6 The relationships between the prognostic model and infiltration of six types of immune cells (B cells, CD4+ T cells, CD8+ T cells, neutrophils, macrophages, and dendritic cells), which are estimated by TIMER algorithm. **Figure S2**, ARHGAP32; **Figure S3**, KLF5; **Figure S4**, MAMLD1; **Figure S5**, MATN3; **Figure S6**, NES.
