# Corrigendum: A Weighted Gene Co-Expression Network Analysis–Derived Prognostic Model for Predicting Prognosis and Immune Infiltration in Gastric Cancer

**DOI:** 10.3389/fonc.2021.683333

**Published:** 2021-04-14

**Authors:** Qingchuan Chen, Yuen Tan, Chao Zhang, Zhe Zhang, Siwei Pan, Wen An, Huimian Xu

**Affiliations:** Department of Surgical Oncology, The First Affiliated Hospital of China Medical University, Shenyang, China

**Keywords:** gastric cancer, weighted gene co-expression network analysis, Cox proportional hazards regression model, prognostic model, immune infiltration

In the original article, there was a mistake in **Figure 9** as published. We made a mistake in the bottom picture of **Figure 9B** due to the disorder in organizing the pictures. The corrected **Figure 9** appears below.

**Figure 9:**
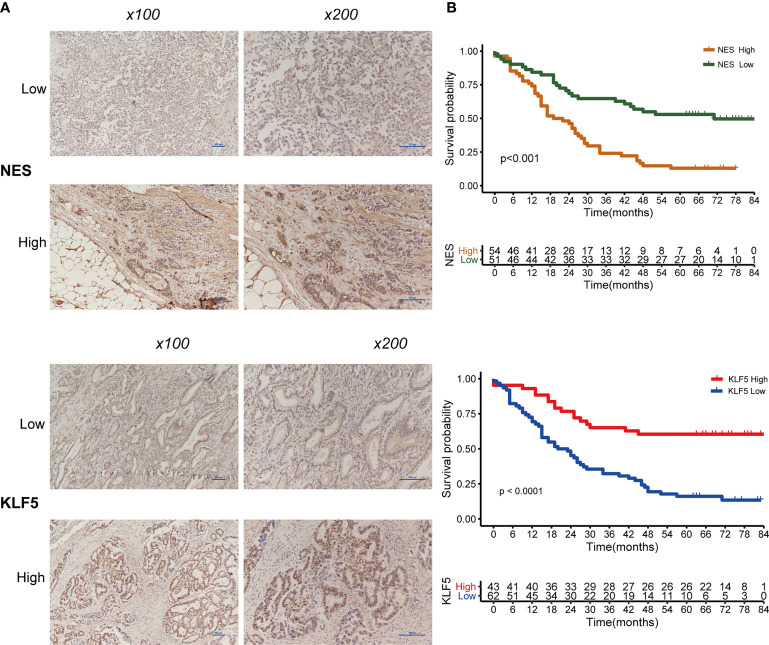
Expression of NES and KLF5 in gastric cancer (GC) tissue and Kaplan-Meier analysis. **(A)** Low and high expression of NES and KLF5 in GC specimens; **(B)** Kaplan-Meier survival curves based on NES and KLF5.

The authors apologize for this error and state that this does not change the scientific conclusions of the article in any way. The original article has been updated.

