# Supplementary figures and images for: A Weighted Gene Co-Expression Network Analysis–Derived Prognostic Model for Predicting Prognosis and Immune Infiltration in Gastric Cancer
Source: Front Oncol. 2021 Feb 25;11:554779. doi: 10.3389/fonc.2021.554779 (PMC7947930; doi:10.3389/fonc.2021.554779)

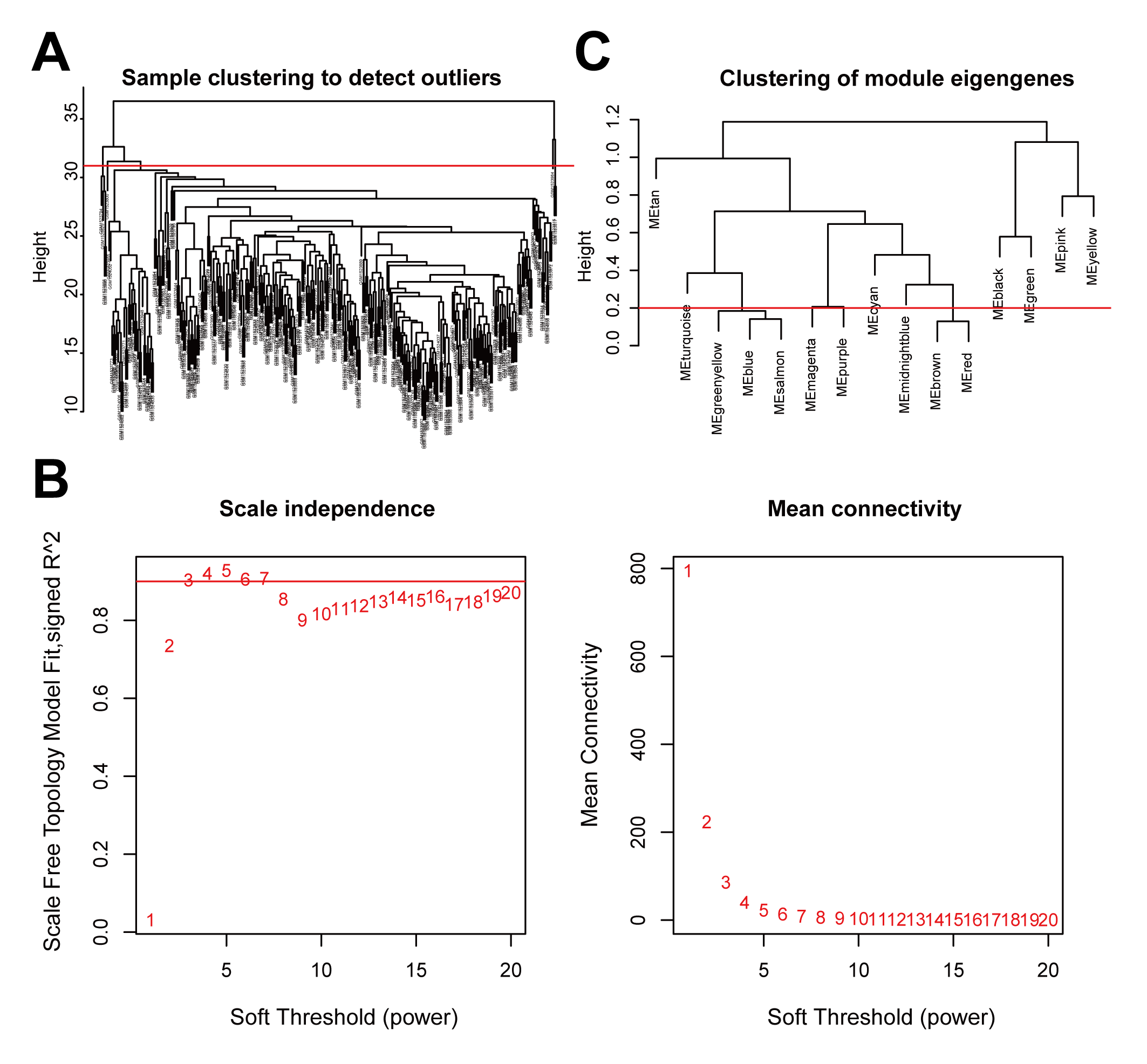

Supplement: Supplementary Figure 1 — (A) Sample cluster dendrogram. Red line represents height of 31. (B) Analysis of scale-free independence and mean connectivity to select optimal soft-threshold power. (C) Clustering of MEs. The red line represented height of 0.2. ME, module eigengenes. [file Image_1.tif]

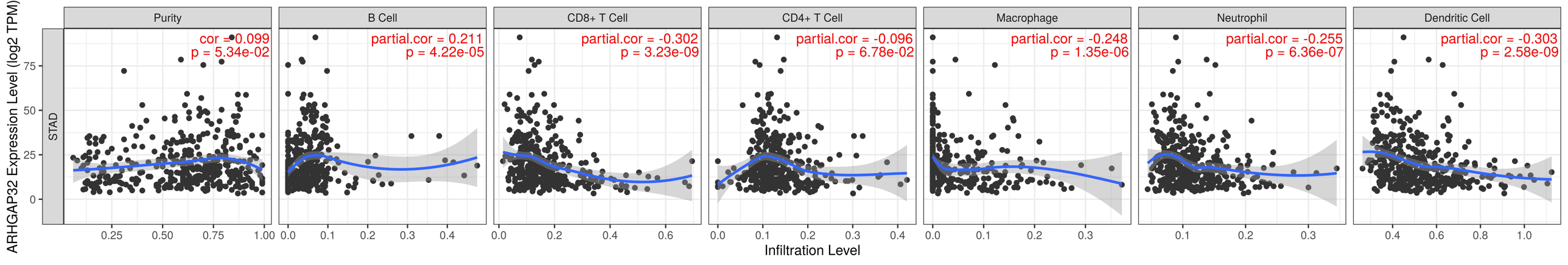

Supplement: Supplementary Figure 2 — -S6 The relationships between the prognostic model and infiltration of six types of immune cells (B cells, CD4+ T cells, CD8+ T cells, neutrophils, macrophages, and dendritic cells), which are estimated by TIMER algorithm. Figure S2 , ARHGAP32; Figure S3 , KLF5; Figure S4 , MAMLD1; Figure S5 , MATN3; Figure S6 , NES. [file Image_2.jpeg]

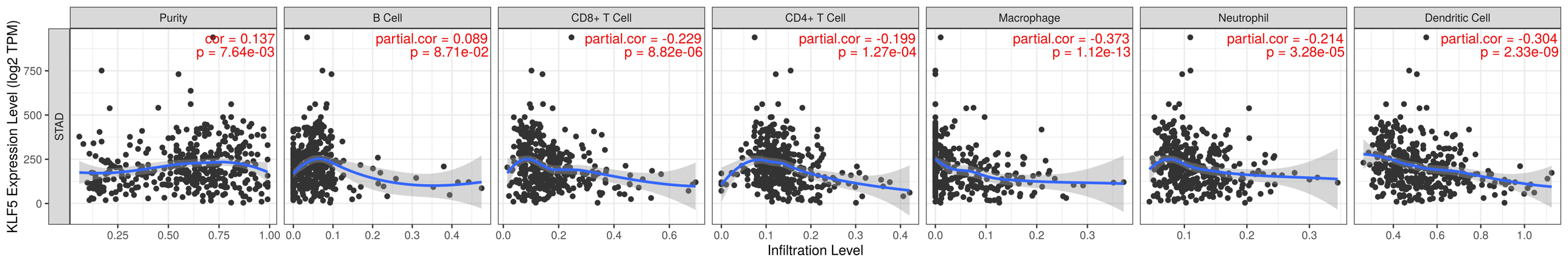

Supplement: Supplementary file 3 [file Image_3.jpeg]

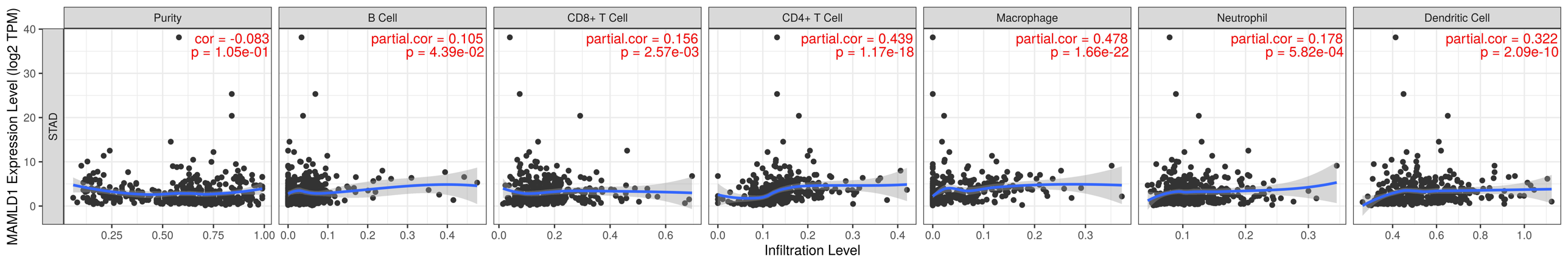

Supplement: Supplementary file 4 [file Image_4.jpeg]

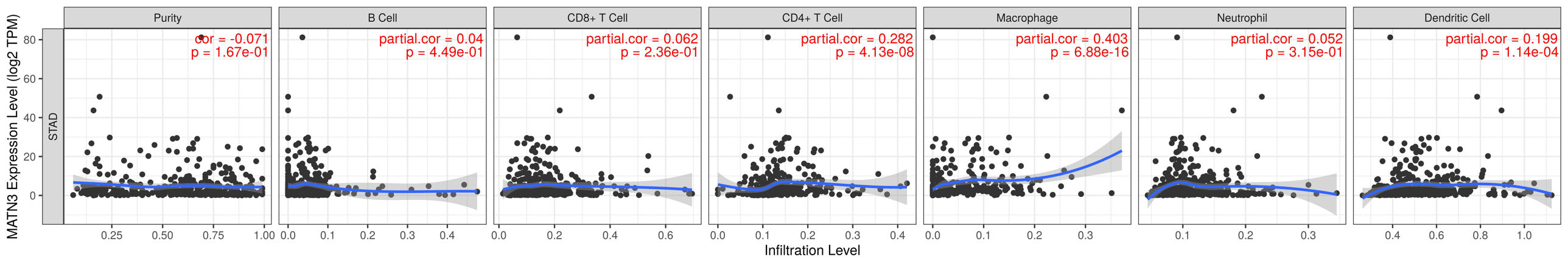

Supplement: Supplementary file 5 [file Image_5.jpeg]

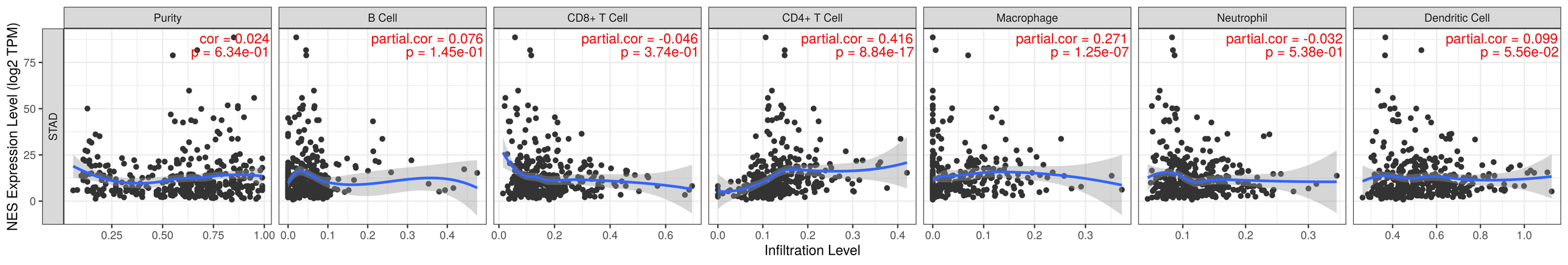

Supplement: Supplementary file 6 [file Image_6.jpeg]
